# Developing a GC-EI-MS/MS method for quantifying warfarin and five hydroxylated metabolites generated by the Fenton reaction

**DOI:** 10.1007/s11356-024-32133-3

**Published:** 2024-02-08

**Authors:** Wipert Jannik von Törne, Urszula-Anna Klyk-Seitz, Christian Piechotta

**Affiliations:** 1 Bundesanstalt für Materialforschung und –prüfung (BAM), Richard-Willstätter-Straße 11, 12489 Berlin, Germany; 2 Technischen Universität Berlin, Straße des 17. Juni 135, 10623 Berlin, Germany

**Keywords:** Fenton reaction, Multiple reaction monitoring, Warfarin, Hydroxy warfarin, Gas chromatography, Quantification

## Abstract

Since the 1950s, Warfarin has been used globally as both a prescription drug and a rodenticide. Research has shown that warfarin and other rodenticides are present in the environment and food chain. However, emerging contaminants are subject to degradation by biotic and abiotic processes and advanced oxidation processes. In some cases, detecting the parent compound may not be possible due to the formation of structurally changed species. This approach aims to identify hydroxylated transformation products of warfarin in a laboratory setting, even after the parent compound has undergone degradation. Therefore, the Fenton reaction is utilized to insert hydroxylation into the parent compound, warfarin, by hydroxyl and hydroperoxyl radicals generated by Fe^2+^/Fe^3+^ redox reaction with hydrogen peroxide. Using multiple reaction monitoring, a GC–MS/MS method, incorporating isotopically labeled reference compounds, is used to quantify the expected derivatized species. The analytes are derivatized using trimethyl-3-trifluoromethyl phenyl ammonium hydroxide, and the derivatization yield of warfarin is determined by using isotopically labeled reference compounds. The method has a linear working range of 30 to 1800 ng/mL, with detection limits ranging from 18.7 to 67.0 ng/mL. The analytes are enriched using a C18-SPE step, and the recovery for each compound is calculated. The Fenton reaction generates all preselected hydroxylated transformation products of warfarin. The method successfully identifies that 4′-Me-*O*-WAR forms preferentially under the specified experimental conditions. By further optimizing the SPE clean-up procedures, this GC–MS-based method will be suitable for detecting transformation products in more complex matrices, such as environmental water samples. Overall, this study provides a better understanding of warfarin’s degradation and offers a robust analytical tool for investigating its transformation products.

**Supplementary Information:**

The online version contains supplementary material available at 10.1007/s11356-024-32133-3.

## Introduction

Link et al. synthesized the vitamin K epoxide reductase inhibitor warfarin (WAR) based on their findings of the sweet clover disease (Campbell & Link 1941; Link 1959). WAR interacts with the vitamin K-depending activation of several blood clotting precursors in the blood coagulation cascade (Rishavy et al. 2011; Stafford 2005). The anticoagulant therapeutic WAR is still a commonly administered drug in preventing pulmonary embolism, thrombosis, atrial fibrosis, and fibrillation (Link 1959; Wardrop & Keeling 2008). The global market size of anticoagulants is estimated to increase to $ 45.50 billion by 2026, according to Fortune Business Insights’ report (Anticoagulants Market Size, Share & Industry Analysis, By Disease Indication (Pulmonary Embolism (PE), Deep Vein Thrombosis (DVT), Atrial Fibrillation, Heart Attacks, Others), By Route of Administration (Oral, Injectable), By Distribution Channel (Hospitals Pharmacies, Retail Pharmacies, Online Pharmacies, Others) and Regional Forecast, 2019–2026.) (https://www.fortunebusinessinsights.com/industry-reports/anticoagulants-market-101807, 2019). That reflects the therapeutic market’s uptake in usage but not the biocide market. warfarin remains in excrement, sewage water, and wastewater treatment plants when administered. The application of rat poison in the form of bait is of particular concern to the environment. Consumed from non-target animals via deceased target species and from bait systems, warfarin releases into the environment (Regnery et al. 2020). Numerous publications exist on warfarin and other anticoagulant rodenticides (ARs) in targeted and untargeted wildlife species and domesticated animals (Nakayama et al. 2019; Rattner et al. 2014; Waddell et al. 2013). ARs have been detected in agricultural products (Saito-Shida et al. 2016) and wastewater (Fernandez et al. 2014; Gomez-Canela et al. 2014) and aquatic environments (Regnery et al. 2018; Regnery et al. 2019). Indeed, no method takes advantage of the known metabolites of warfarin for its analytical detection. Pharmacologically, warfarin is primarily eliminated via the hepatic phase 1 metabolism and urinary excretion. In humans, cytochrome P450 (CYP) enzymes selectively transform warfarin stereo into enantiomers of 6-, 7-, and 8-hydroxy warfarin (Kaminsky & Zhang 1997). Stereoisomers of dehydro-warfarin, 4′-hydroxy warfarin, and 10-hydroxy warfarin also yield from this biotransformation and warfarin alcohol (Kaminsky & Zhang 1997). These metabolites are not unique to mammalian species (Watanabe et al. 2015). Warfarin’s metabolism in rodents, birds, and other species is well documented (Kaminsky & Zhang 1997; Rettie et al. 1992; Saengtienchai et al. 2011; Watanabe et al. 2015). CYPs generally mediate reactions via hydrogen atom transfer (HAT) and single-electron transfer (SET) (Meunier et al. 2004).

**Fig. 1:**
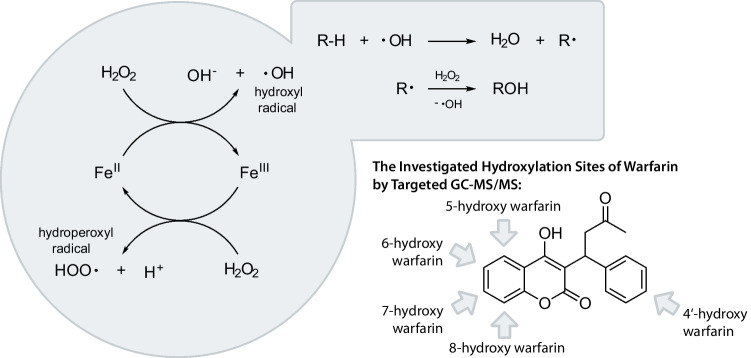
Schematic overview of the Fenton reaction and the hydroxylation of organic substances by the reaction with hydroxyl radicals and Fenton’s reagent and the hydroxylation sites of warfarin investigated in this study by targeted GC–MS/MS

One prominent example of organic matter and reactive-oxygen-species (ROS) reactions is the Fenton Reaction, which is an effective advanced oxidation process (AOP) with applications in wastewater treatment (Nidheesh & Gandhimathi 2012; Pignatello et al. 2006; Zhang et al. 2019). This reaction has attracted considerable attention due to its exceptional ability to degrade various organic pollutants effectively (Gligorovski et al. 2015; Miklos et al. 2018; Nidheesh & Gandhimathi 2012; Sharma et al. 2018). The Fenton reaction offers an efficient solution to tackle the growing challenge of wastewater contamination caused by various industrial, agricultural, and domestic activities (Gligorovski et al. 2015; Miklos et al. 2018; Nidheesh & Gandhimathi 2012; Sharma et al. 2018). The reaction can lead to complete mineralization by oxidation of organic matter (Nidheesh & Gandhimathi 2012; Zhang et al. 2019). The “oxidation of tartaric acid in the presence of iron” was the first reaction demonstration by H. J. H. Fenton in 1894 (Fenton 1894). Fenton’s reagent is the Haber–Weiss-like (Kehrer 2000; Koppenol 2001) reaction between hydrogen peroxide (H_2_O_2_) and ferrous iron in an acidic solution (Brillas et al. 2009). The pH optimum is about 2.8 and 3 (Brillas et al. 2009). During the reaction, Fe^2+^ is oxidized to Fe^3+^, while hydrogen peroxide reacts to hydroxide anions (OH^−^) and hydroxyl radicals (∙OH). Figure 1 illustrates this reaction. Formed radicals can react further with organic compounds, possibly leading to complete mineralization. During Fenton’s reaction, various radical species form, which can react with hydrogen peroxide, generating hydroxylated transformation products. Since hydroxylated products are expected to be formed during the reaction of WAR and Fenton’s reagent, their detection and quantification are particularly interesting for monitoring environmental samples and wastewater treatment. Regarding WAR, a hydroxylation in different positions is plausible. Figure 1 summarizes the investigated hydroxylation sites in this study.

A few GC-based analytical methods from the nineteen hundreds showed the quantification of warfarin and its known metabolites. An overview is presented in this paragraph. Numerous studies have quantified hydroxylated warfarin species using liquid chromatography and mass spectrometry (Kim et al. 2012; Spink et al. 1989; Watanabe et al. 2015). Gas chromatographic techniques have been less frequently employed, with notable methods dating back to the 1970s and 1990s. Kaiser et al. (Kaiser & Martin 1974) and Midha et al. (Midha et al. 1974) reported the first gas chromatography-based quantification methods for warfarin in plasma, utilizing pentafluorobenzyl-derivative utilizing Tracor MT-220 GC-ECD. In 1978, Hanna et al. (Hanna et al. 1978) obtained a similar detection limit employing GC-FID. Duffield et al. (1979a, b) quantified methylated warfarin and its alcohol using deuterated internal standards (A. M. Duffield et al. 1979a, b). Davies et al. (Davies et al. 1983) published a direct insertion method with a 200 ng/mL detection limit. Bush et al. (Bush et al. 1983) reported a detection limit of 1 ng/mL for methylated warfarin. Maurer et al. (Maurer & Arlt 1998) detected 4-hydroxy coumarins in urine using GC–MS. In 2008, Sato demonstrated SIM quantification of warfarin in human serum using GC-EI-MS with a reported determination limit of 20 ng/mL after TMS-DAM derivatization (Sato 2005). Most of the gas chromatographic methods mentioned here have been used to determine warfarin in plasma samples. However, since most of these methods do not correspond to contemporary technology, there is a variety of, e.g., liquid chromatography-based methods that were successfully applied for determining warfarin’s metabolites in blood samples recently summarized by Mulyadi et al. (Mulyadi et al. 2023). This study aims to develop and assess the efficacy of a contemporary GC–MS/MS method designed to identify and quantify targeted warfarin metabolites and transformation products.

## Experimental section

Analytical grade warfarin (4-hydroxy-3-(3-oxo-1-phenyl butyl)chromen-2-one) (PESTANAL®, analytical standard, Fluka, Sigma-Aldrich), 4′-hydroxy warfarin (4-hydroxy-3-(1-(4-hydroxyphenyl)-3-oxobutyl)chromen-2-one) (Sigma-Aldrich), 6-hydroxy warfarin (4,6-dihydroxy-3-(3-oxo-1-phenyl butyl)chromen-2-one) (Chemos GmbH Co. KG, Altdorf, Germany), 7-hydroxy warfarin (4,7-dihydroxy-3-(3-oxo-1-phenyl butyl)chromen-2-one) (Chemos GmbH Co. KG, Altdorf, Germany), and 8-hydroxy warfarin (4,8-dihydroxy-3-(3-oxo-1-phenyl butyl)chromen-2-one) (Chemos GmbH Co. KG, Altdorf, Germany) reference standards were used in this study for methods development. ASCA GmbH (Angewandte Synthesechemie Adlershof, Berlin, Germany) synthesized 5-hydroxy Warfarin (4,5-dihydroxy-3-(3-oxo-1-phenyl butyl) chromen-2-one) and the isotopically labeled internal standards: phenyl ^13^C_6_-isotopomers of 5-hydroxy Warfarin, 6-hydroxy Warfarin, Me^13^C-WAR (4-(^13^C)methoxy-3-(3-oxo-1-phenyl butyl)chromen-2-one), and 7-hydroxy Warfarin. Phenyl D_5_ warfarin (4-hydroxy-3-(3-oxo-1-(2,3,4,5,6-pentadeuteriophenyl) butyl) chromen-2-one) (Dr. Ehrenstorfer GmbH, Augsburg, Germany) was commercially available. For methods’ development and validation, standard stock solutions of all 4-hydroxy coumarins were prepared separately in MS-grade acetonitrile (Biosolve BV, Valkenswaard, The Netherlands) to obtain a final concentration of 0.1 μg/μL. Mixed stock solutions of the non-labeled reference standards were composed of the standard stock solutions and were diluted to a concentration of 2.0 ng/μL. Internal standard stock solutions of the isotopically labeled standards were prepared accordingly.

Degradation of warfarin undergoing the Fenton Reaction was performed in Erlenmeyer flasks in a sulfate buffer at pH 3.0 at room temperature. The initial concentration of warfarin was 1.25 μg/mL (1250 ng/mL). A stock solution of ferrous sulfate heptahydrate (96 mg/mL) p. a. (CHEMSOLUTE®, Th. Geyer GmbH & Co. KG, Renningen, Germany) in sulfate buffer and hydrogen peroxide 30% (CHEMSOLUTE®) was added in a molar ratio of 1/1/50//WAR/Fe^2+^/H_2_O_2_ to start the reaction. Quenching after 2, 4, 6, 8, 16, 30, and 60 min results in independent samples, and adding 2 mL 0.1 m sodium hydroxide solution (J.T.Baker) per 100 mL buffer led to the precipitation of ferric salts. Additionally, radical species were quenched by adding 500 mg sodium thiosulfate (anhydrous, Merck KGaA, Darmstadt, Germany). One milliliter of the solution was taken for the quantification of warfarin. SPE was used to concentrate the samples in a 1:1 ratio. The remaining solution was also subjected to SPE to quantify hydroxylated species and concentrated in a 1:100 ratio.

Matrix separation and sample concentration were achieved using BAKERBOND™ C_18_ solid phase extraction (SPE) cartridges (6 mL, 500 mg C_18_-bound silica gel, 40 μm APD, 60 Å, J.T. Baker). SPE cartridges were washed with approximately 6 mL acetonitrile and preconditioned with the same amount of deionized H_2_O. After adding the internal standard stock solution, the neutralized (pH 6–7) samples were loaded onto the cartridge’s material and passed through by applying a vacuum. The SPE material was rinsed with approximately 6 mL of deionized water and left to dry. The analytes were eluted in 5–6 mL of acetonitrile and evaporated to dryness under a stream of nitrogen at 40 °C. The recovered solid was redissolved in acetonitrile and prepared for GC-EI-MS/MS analysis.

For GC-EI-MS/MS measurements, 350 μL of the samples was mixed with 50 μL of the derivatization agent, *m*-TFPTAH, and trimethyl-3-trifluoromethyl phenyl ammonium hydroxide 5% in methanol (abcr GmbH, Karlsruhe, Germany; and Alfa Aesar, Thermo Fischer GmbH, Kandel, Germany). The samples were subjected to Shimadzu’s TQ8040 GC–MS/MS. One microliter was injected by splitless injection. The injector temperature was set to 270 °C for in-liner methylation of hydroxyl groups. A helium flow rate of 1 mL/min was used for chromatographic separation on a DB5 MS-UI 30 m × 0.25 mm × 0.25 μm (Agilent Technologies) fused silica capillary column using the following oven program: 100 °C → 280 °C (22 K/min) → 320 °C (7 K/min, 1.1 min hold 320 °C). The transfer line was heated to 280 °C, and the ion source temperature was set to 240 °C. Ionization was achieved by standard electron energy of 70 eV and 5.0 μA emission current at 200 °C, relative to the latest used tune file.

## Results and discussion

### GC–MS/MS methods development

**Fig. 2:**
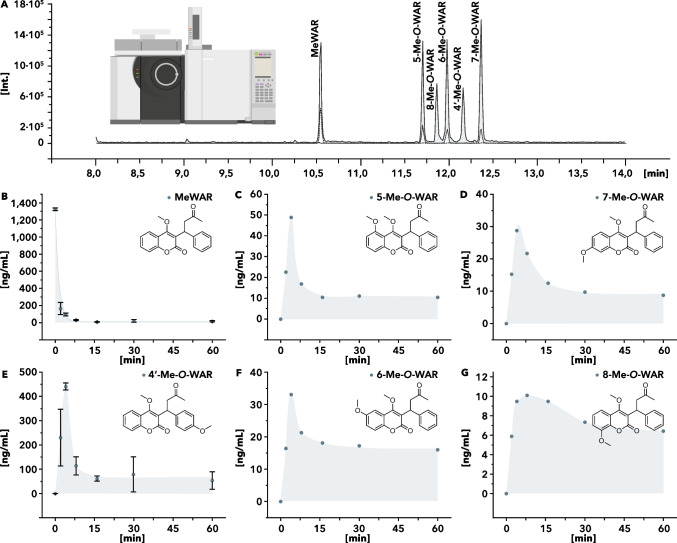
**A** Total-ion-chromatogram of the reference compounds and isotopically labeled reference compounds and **B**–**G** overview of warfarin’s degradation and the formation of hydroxylated transformation products during the Fenton reaction. **A** exemplifies a total-ion-chromatogram of the reference substances. The peaks of the standard substances are shaded in light gray, and those of the corresponding isotopically labeled standard substances with lower intensities are backgrounded in white. **B** shows the results of the degradation of warfarin. Additionally, the figure illustrates the formation and degradation of **C** 5-Me-*O*-WAR, **D** 7-Me-*O*-WAR, **E** 4′-Me-*O*-WAR, **F** 6-Me-*O*-WAR, and **G** 8-Me-*O*-WAR

Optimization of the GC parameters achieved baseline separation of the analytes. In this study, hydroxyl group alkylation utilizing m-TFPTAH was used to enhance the thermal stability and volatility of the analytes, aligning with the recommended injector temperature conditions outlined in the literature (Brombacher et al. 1977; Kossa et al. 1979; Midha et al. 1976). The etherification processes were conducted in the injection port within the liner at elevated temperatures. In the precursor ion scan, specific target ions confirm the analytes’ identity by collision-induced-dissociation. Figure 2 A shows a total-ion-chromatogram of the reference compounds and isotopically labeled reference compounds. Table 1 comprises the qualifier and quantifier ions, the optimized transitions, and the retention times of the analytes. An overview of the optimization process of the transitions and collision energies (CE) of the selected product ions is shown in the supporting information in Figure and Table 1–10. The quantification of MeWAR was monitored using the transition of the M^+^-43 cation (*m/z* 279, C_18_H_15_O_3_^+^) to *m/z* 128 (C_10_H_8_^+^). The same fragment with shifted *m/z* values was selected for the phenyl D_5_-labelled internal standard (Me-D_5_WAR). 4′-Me-*O*-WAR was determined utilizing *m/z* 128, too. But with a different precursor ion (M^+^-43, *m/z* 309, C_19_H_17_O_4_^+^). 7- and 5-Me-*O*-WAR were determined using the generation of *m/z* 227 (C_18_H_13_O_3_^+^) from the M^+^-43 cation (*m/z* 309, C_19_H_17_O_4_^+^). Corresponding *m/z* values of the phenyl ^13^C_6_-labelled species are listed in Table 1. The transition of the radical cation (*m/z* 352, C_21_H_20_O_5_^•+^) to the M^+^-43 cation (*m/z* 309, C_19_H_17_O_4_^+^) was used for the quantification of 6- and 8-Me-*O*-WAR. Because of the similar fragmentation behavior of 6-, 7-, and 8-Me-*O*-WAR, their qualifier and quantifier ions have identical *m/z* values. Nonetheless, the analytes are uniquely identified based on their retention times and by ^13^C-labeled internal standards. After derivatization, the optimized MRM method ensures high specificity for detecting warfarin and hydroxylated species.

**Table 1:** Overview of the qualifier and quantifier ions, retention times, optimized MRM transitions, and corresponding collision energies of all analytes, including the internal standards. Bold numbers highlight the transition states used for quantification

Analyte	Retention time [min]	MRM-transition			Internal Standard	MRM-transition		
		Ion transition	CE	ratio		ion transition	CE	ratio
MeWAR	10.59	**279.0 → 128.2**	25	89.4	Me-D_5_WAR	**284.0 → 129.2**	25	100.0
		279.0 → 202.1	25	60.7		284.0 → 206.2	25	46.5
		91.0 → 65.1	20	100.0		96.0 → 68.1	20	70.0
4′-Me-*O*-WAR	12.21	**309.0 → 128.2**	25	100.0	Me-D_5_WAR			
		201.0 → 128.2	20	83.2				
		121.0 → 65.1	20	47.4				
5-Me-*O*-WAR	11.71	**309.0 → 277.1**	15	100.0	5-Me-***O***-^13^C_6_WAR	**315.0 → 283.1**	15	100.0
		277.1 → 234.1	20	69.6		283.0 → 240.1	20	71.4
		91.0 → 65.1	20	64.6		97.0 → 69.1	20	41.7
6-Me-*O*-WAR	11.98	309.0 → 277.1	15	62.3	6-Me-***O***-^13^C_6_WAR	315.0 → 283.1	15	70.1
		**352.0 → 309.0**	15	72.5		**358.0 → 315.1**	15	100.0
		91.0 → 65.1	20	100.0		97.0 → 69.1	20	77.0
7-Me-*O*-WAR	12.39	**309.0 → 277.1**	15	46.8	7-Me-***O***-^13^C_6_WAR	**315.0 → 283.1**	15	60.8
		352.0 → 309.1	15	59.0		358.0 → 315.1	15	79.0
		91.0 → 65.1	20	100.0		97.0 → 69.1	20	100.0
8-Me-*O*-WAR	11.87	309.0 → 277.1	15	60.6	6/7- Me-***O***-^13^C_6_WAR			
		**352.0 → 309.1**	15	74.6				
		91.0 → 65.1	20	100.0				

The mixed stock solution of non-labeled reference standards was used to prepare calibration levels with concentrations ranging from 30 to 1800 ng/mL. Due to the extended working range, 15 calibration points were chosen to ensure linearity. In the decade 30 to 300 ng/mL, ten calibration points were set in 30 ng/mL intervals. From 300 to 1800 ng/mL, 5 points were selected with intervals of 300 ng/mL each. The concentration of the IS in these samples was 200 ng/mL. Trimethyl-3-trifluoromethyl phenyl ammonium was added to the samples in a volumetric ratio of 1:7. Therefore, the linearity of the working range was determined in a final concentration range of 26 to 1543 ng/mL. The final concentration of the IS was 171.4 ng/mL. Data were processed by plotting the obtained peak areas relative to the corresponding peak area of the internal standard versus the analyte’s concentration. After linear regression, slopes are significantly different from zero. Table 2 summarizes the obtained values. The ratios of the peak areas are directly proportional to the concentration of the analytes. The coefficients of determination (COD) of all analytes are shown in Table 2. Apart from 6-Me-*O*-WAR, all coefficients are higher than 0.9965. The coefficient of 6-Me-*O*-WAR is within the acceptance criteria at 0.9947. Warfarin shows the highest correlation with 0.9994. Data evaluation proves a linear relationship within the defined working range for all analytes.

**Table 2:** Overview of the obtained slopes of the regression, y-intercept, and coefficients of determination of all analytes, including the detection limits calculated according to DIN 32645 (15 standard levels with three injections) and SPE recovery rates

Analyte	The slope of the regression line [ng/mL]	y-intercept	*R*^2^ (COD)	LOQ [ng/mL]	LDL [ng/mL]		LOD [ng/mL]	SPE recovery rates	
								Absolute [%]	Relative [%]
MeWAR	6.01·10^−3^ ± 3.9·10^−5^	0.0342 ± 0.0281	0.9994	81.5		48.7	24.4	97.1 ± 2.3	97.5 ± 1.3
4′-Me-*O*-WAR	6.14·10^−3^ ± 9.7·10^−5^	− 0.1622 ± 0.0801	0.9965	125.3		76.2	38.1	90.4 ± 1.4	108.3 ± 0.9
5-Me-*O*-WAR	6.09·10^−3^ ± 7.9·10^−5^	0.0523 ± 0.0666	0.9980	60.7		37.3	18.7	37.0 ± 8.5	75.0 ± 2.3
6-Me-*O*-WAR	4.39·10^−3^ ± 8.6·10^−5^	0.1630 ± 0.0670	0.9947	218.2		134.0	67.0	88.6 ± 4.0	94.3 ± 0.6
7-Me-*O*-WAR	5.27·10^−3^ ± 6.9·10^−5^	0.0439 ± 0.0535	0.9978	147.1		89.3	44.7	88.1 ± 1.3	102.6 ± 2.0
8-Me-*O*-WAR	4.20·10^−3^ ± 4.3·10^−6^	0.0340 ± 0.0334	0.9986	110.4		66.1	33.0	94.3 ± 8.4	99.6 ± 3.6

Me^13^C-WAR and MeWAR were analyzed in scan mode in a final concentration of 1 mM in acetonitrile. Integration of the TICs and ion traces at *m/z* 279 (WAR) and *m/z* 280 (Me^13^C-WAR) equal a mean derivatization yield of 33.2 ± 0.3%. However, determining the other compounds’ derivatization yield is not possible in this study since the isotopic label is located within the phenyl ring and not the methyl group. The detection limits were determined using a threefold determination of the calibration standards in the lower working range. The linearity, the limit of quantification (LOQ), the lower limit of detection (LDL), and the limit of detection (LOD) were calculated using the DIN 32645:2008–11 standard of the German Institute for Standardization (DIN). Table 2 summarizes the found values. The lowest LOQ was determined at 60.7 ng/mL for 5-Me-*O*-WAR. Warfarin’s LoQ is 81.5 ng/mL, and 4′-, 7-, and 8-Me-*O*-WAR show a LOQ spanning from 110.4 to 147.1 ng/mL. 6-Me-*O*-WAR showed the highest LOQ at 218.2 ng/mL. The LODs of the analytes ranged from 18.7 to 67.0 ng/mL. Regarding warfarin, the calculated detection limits show dimensions similar to those provided by Sato et al. (Sato 2005).

Six replicate samples of each compound were analyzed to examine the accuracy. For all analytes, a good accuracy of 98.7 ± 2.2% (MeWAR), 102.4 ± 3.2% (4′-Me-*O*-WAR), 101.0 ± 2.6% (5-Me-*O*-WAR), 97.5 ± 2.9% (6-Me-*O*-WAR), 99.7 ± 3.0% (7-Me-*O*-WAR), and 100.6 ± 2.8% (8-Me-*O*-WAR) was obtained. The SPE recovery rate of the analytes was determined in triplicates of 3 different calibration points at 257, 771, and 1286 ng/mL. The samples were subjected to SPE and analyzed by GC–MS/MS. Reference compounds and the IS were transferred to a 5-mL volumetric flask, dried, and redissolved in 5 mL of neutralized (pH 6–7) sulfate buffer to determine the relative recovery. The absolute recovery rate was determined by adding the IS after SPE. Table 2 summarizes the mean values of the relative and absolute recovery rates comprising all chosen calibration points. The mean recoveries determined are all within the acceptance criteria at 80–120%. Only 5-Me-*O*-WAR shows a poor recovery. The relative recovery is about 75%, whereas the absolute recovery is 37%. Due to the hydroxyl groups’ parallel arrangement and spatial proximity in 5-OH-WAR, it is well suited for chelating metal ions. Such chelating properties have been reported for flavonoids (Riha et al. 2014) as well as antioxidant and free radical scavenging activity regarding the coumarin moiety (Annunziata et al. 2020). Solubility factors likely contribute to the concentration of 5-OH-WAR as a hydrophilic metal-complex might result, which may not be adequately retained on the SPE-cartdridge’s material. In general, it appears that the SPE method applied in this study is less suitable for 5-OH-WAR.

### Aqueous degradation of warfarin

The Fenton reaction requires hydrogen peroxide and dissolved ferric salts. The influence of hydrogen peroxide on the system was examined in the early stages of the experiment’s design. Results proved that the buffer’s pH value and the warfarin concentration do not change due to H_2_O_2_. Furthermore, test measurements showed that hydrogen peroxide does not lead to a degradation of warfarin. After the reaction, sodium thiosulfate was used to quench radical species. During initial tests (data not shown) that used HPLC–DAD detection, ascorbic acid was utilized as a quencher. However, these tests showed no definite correlation between the amount of hypochlorite used and the decrease in warfarin. The degradation appeared to be random and not reproducible. The hypothesis was formed that the quencher may not have been sufficient to terminate the reaction. Therefore, sodium thiosulfate was used instead of ascorbic acid. This change resulted in reproducible and interpretable outcomes. In addition, 0.1 M sodium hydroxide solution was used to stop the Fenton reaction due to a shift to alkaline pH. The required amount was determined experimentally by observing the change in pH while adding different amounts of sodium hydroxide solution to the buffer. N. Wang et al. reported an equation calculating the minimum molar equivalents of hydrogen peroxide needed for complete degradation. It uses the sum formula following: C_a_H_b_N_c_O_d_ (Wang et al. 2016). Using the given Eq. (2a + 1/2b + 5/2c -d) on warfarin’s sum formula C_19_H_16_O_4_, 42 results are needed as molar equivalents for complete degradation. Accordingly, 1:50 defines the minimum molar equivalents of warfarin and hydrogen peroxide in the following experiments to achieve complete analyte degradation.

Degradation of aqueous WAR was achieved as described in the “Experimental section.” The ratios of the peak areas of the analytes and their internal standards were determined to calculate the concentration of each analyte at distinct time points. Figure 2 B–G illustrate the results. Additionally, areas highlighted in light gray visualize the change in the analyte’s concentration. Figure 2 B shows the aqueous degradation of warfarin. The degradation was fitted and provided a COD of 0.9985. Complete degradation of WAR was achieved within the first 10 min. Figure 2 C–G summarize the calculated amount of hydroxylated TPs during the Fenton reaction. All targets were detected and showed a maximum concentration after approximately 4 min, followed by decreasing concentrations. As described in the “Experimental section,” 1 mL of the independent samples was used to determine MeWAR and 4′-Me-*O*-WAR simultaneously with the other analytes.

The concentration of MeWAR and 4′-Me-*O*-WAR was determined in triplicates to ensure that these samples were representative. Samples were taken in a total volume of 1 mL at the indicated time points to observe their degradation behavior in separate samples. In the case of MeWAR and 4′-Me-*O*-WAR, all the obtained data were merged and plotted with their standard deviations. Analysis of the other analytes was performed as described. Among the investigated hydroxylated TPs, 8-Me-*O*-WAR is quantitatively formed at the lowest level. The maximum intensity was recorded after approximately 8 min with 10 ng/mL. The concentration subsequently decreased again. Regarding the other analytes, the maximum intensities were reached at around 4 min and immediately reduced after that. Approximately three times the amount of 8-Me-*O*-WAR was detected for 6-Me-*O*-WAR and 7-Me-*O*-WAR. For these analytes, the maxima are 33 ng/mL (6-Me-*O*-WAR) and 29 ng/mL (7-Me-*O*-WAR). It was possible to detect 49 ng/mL of 5-Me-*O*-WAR after a reaction time of 4 min. At 441 ng/mL, 4′-Me-*O*-WAR was generated with the highest quantity. To further relate the obtained data to one another, the percentages of the concentrations were calculated using their molar masses relative to the initial concentration of warfarin. WAR’s initial concentration was reduced from 100 to 7.2% within the first 4 min. After 1 h, 1.2% remained. The hydroxylated species were formed within the same period. After 4 min, the overall peak concentration was 38.8% and 6.6% of the determined TPs were detected after one hour. 4′-OH-WAR was generated in much higher quantities than the other targeted TPs. Its maximum yield was 30.4%. After 1 h, its concentration decreased to 3.7%. 5-, 6-, 7- and 8-OH-WAR have much lower yields than 4′-OH-WAR. The percentage of hydroxylated TPs at the coumarin moiety ranges from 8.3% after 4 min to 2.9% after 1 h of reaction time. After 4 min, the peak concentration of these analytes was determined in the range of 3.4% (5-OH-WAR) and 0.7% (8-OH-WAR). After 1 h, 1.1% (6-OH-WAR) and 0.4% (8-OH-WAR) were calculated. The formation of a steady-state concentration suggests that the reaction will potentially stop due to the consumption of hydrogen peroxide.

In 1968, Masayuki et al. found via thin-layer-chromatography that 6-OH-WAR and 8-OH-WAR are predominantly formed during the Fenton reaction, while the amount of 7-OH-WAR formed was relatively small (Masayuki et al. 1968). Respectively to the used ratios of the reagents, it is shown that 4-OH-WAR is formed most preferably in this study. The 4′ position of the phenyl ring is highly reactive to a radical attack at the activated and sterically accessible *para*-position of the phenyl ring. In addition, 5-, 6-, and 7-OH-WAR are formed similarly. 5-OH-WAR shows the highest intensity. That is followed by 6- and 7-OH-WAR. At the same time, 8-OH-WAR is formed only slightly as an intermediate. As the reaction proceeds, 5-, 7-, and 8-OH-WAR are found in similar amounts. 6-OH-WAR appears to be marginally more stable. Although 4′-OH-WAR is most abundant after 1 h, its abundance decreases by a power of ten within the first 15 min. It can, therefore, be assumed that this form continues to react rapidly. Here, double hydroxylated TPs are likely to occur, as in the case of all other TPs.

## Conclusion

In literature, trimethylsilyl diazomethane was often used for the methylation of warfarin and hydroxylated analogous. This study shows promising results for in-liner methylation with trimethyl-3-trifluoromethyl phenyl ammonium hydroxide. The derivatization yield of warfarin equaled 33.2 ± 0.3% and was calculated using the synthesized isotopically labeled Me^13^C-WAR. Detection and quantification of methylated warfarin species using the established GC-EI-MS/MS method, optimized oven program, and the set ion transitions prove sufficient selectivity. Me-D_5_WAR and phenyl ^13^C_6_-labeled reference compounds were used as internal standards. All analytes show good linearity in the investigated 26 to 1543 ng/mL concentration range, and the calculated limits of detection range between 18.7 and 67.0 ng/mL. Except for 5-hydroxy warfarin, all other investigated species show good SPE recoveries. The method was successfully applied to quantify the decrease in warfarin undergoing the Fenton Reaction. Additionally, the formation and degradation of hydroxylated transformation products at a set molar ratio were successfully monitored. Due to the sterically accessible and highly reactive *para*-position of the phenyl ring, 4′-OH-WAR was preferably formed. The method described in this study is a suitable alternative to the commonly used LC–MS techniques and offers the determination of warfarin based on its mono-hydroxylated metabolites. Possible applications are the determination of warfarin and the described hydroxylated species in the more complex matrices, such as wastewater purification, biological, or environmental samples.

### Supplementary Information

Below is the link to the electronic supplementary material.

Supplementary file1 (PDF 1.62 MB)

## Data Availability

Not applicable.

## References

[CR1] Annunziata F, Pinna C, Dallavalle S, Tamborini L, Pinto A (2020). An Overview of Coumarin as a versatile and readily accessible scaffold with broad-ranging biological activities. Int J Mol Sci.

[CR2] Brillas E, Sires I, Oturan MA (2009). Electro-Fenton process and related electrochemical technologies based on Fenton’s reaction chemistry. Chem Rev.

[CR3] Brombacher PJ, Cremers HMHG, Mol MJ, Muijrers PHJ, Van Der Plas PM, Verheesen PE (1977). A gas chromatographic method for the estimation of phenprocoumon, 3-(1-phenyl-propyl)-4-hydroxycoumarin (marcoumar®, liquamar®), in human serum or plasma. Clin Chim Acta.

[CR4] Bush ED, Low LK, Trager WF (1983). A sensitive and specific stable isotope assay for warfarin and its metabolites. Biomed Mass Spectrom.

[CR5] Campbell HA, Link KP (1941) Studies on the hemorrhagic sweet clover disease IV. The isolation and crystallization of the hemorrhagic agent. J Biol Chem 138(1), 21–33. Retrieved from <<Go to ISI>://WOS:000187900800002>

[CR6] Davies NW, Bignall JC, Roberts MS (1983). Direct quantitative determinations by multiple metastable peak monitoring. 1–Warfarin in plasma. Biomed Mass Spectrom.

[CR7] Duffield AM, Duffield PH, Birkett DJ, Kennedy M, Wade DN (1979). Plasma quantitation of warfarin and warfarin alcohol by gas chromatography chemical ionization mass spectrometry in patients on warfarin maintenance therapy. Biomed Mass Spectrom.

[CR8] Duffield PH, Birkett DJ, Wade DN, Duffield AM (1979). Quantitation of plasma warfarin levels by gas chromatography chemical ionization mass spectrometry. Biomed Mass Spectrom.

[CR9] Fenton HJH (1894) LXXIII.—Oxidation of tartaric acid in presence of iron. J Chem Soc Trans 65(0), 899-910. 10.1039/ct8946500899

[CR10] Fernandez I, Santos A, Cancela ML, Laize V, Gavaia PJ (2014). Warfarin, a potential pollutant in aquatic environment acting through Pxr signaling pathway and gamma-glutamyl carboxylation of vitamin K-dependent proteins. Environ Pollut.

[CR11] Gligorovski S, Strekowski R, Barbati S, Vione D (2015). Environmental implications of hydroxyl radicals ((*)OH). Chem Rev.

[CR12] Gomez-Canela C, Barata C, Lacorte S (2014). Occurrence, elimination, and risk of anticoagulant rodenticides and drugs during wastewater treatment. Environ Sci Pollut Res Int.

[CR13] Hanna S, Rosen M, Eisenberger P, Rasero L, Lachman L (1978). GLC determination of warfarin in human plasma. J Pharm Sci.

[CR14] https://www.fortunebusinessinsights.com/industry-reports/anticoagulants-market-101807 (2019) Anticoagulants market size, share & industry analysis, by disease indication (pulmonary embolism (PE), deep vein thrombosis (DVT), atrial fibrillation, heart attacks, others), by route of administration (oral, injectable), by distribution channel (hospitals pharmacies, retail pharmacies, online pharmacies, others) and regional forecast, 2019–2026. Retrieved from https://www.fortunebusinessinsights.com/industry-reports/anticoagulants-market-101807

[CR15] Kaiser DG, Martin RS (1974). GLC determination of warfarin in human plasma. J Pharm Sci.

[CR16] Kaminsky LS, Zhang ZY (1997). Human P450 metabolism of warfarin. Pharmacol Ther.

[CR17] Kehrer JP (2000). The Haber-Weiss reaction and mechanisms of toxicity. Toxicology.

[CR18] Kim SY, Kang JY, Hartman JH, Park SH, Jones DR, Yun CH, Miller GP (2012). Metabolism of R- and S-warfarin by CYP2C19 into four hydroxywarfarins. Drug Metab Lett.

[CR19] Koppenol WH (2001). The Haber-Weiss cycle - 70 years later. Redox Rep.

[CR20] Kossa WC, MacGee J, Ramachandran S, Webber AJ (1979). Pyrolytic methylation/gas chromatography: a short review. J Chromatogr Sci.

[CR21] Link KP (1959). The discovery of dicumarol and its sequels. Circulation.

[CR22] Masayuki I, Ullrich V, Staudinger H (1968). Metabolism in vitro of warfarin by enzymic and nonenzymic systems. Biochem Pharmacol.

[CR23] Maurer HH, Arlt JW (1998). Detection of 4-hydroxycoumarin anticoagulants and their metabolites in urine as part of a systematic toxicological analysis procedure for acidic drugs and poisons by gas chromatography mass spectrometry after extractive methylation. J Chromatogr B.

[CR24] Meunier B, de Visser SP, Shaik S (2004). Mechanism of oxidation reactions catalyzed by cytochrome p450 enzymes. Chem Rev.

[CR25] Midha KK, Hubbard JW, Cooper JK, McGilveray IJ (1976). GLC determination of plasma concentrations of phenprocoumon. J Pharm Sci.

[CR26] Midha KK, McGilveray IJ, Cooper JK (1974). GLC determination of plasma levels of warfarin. J Pharm Sci.

[CR27] Miklos DB, Remy C, Jekel M, Linden KG, Drewes JE, Hubner U (2018). Evaluation of advanced oxidation processes for water and wastewater treatment - a critical review. Water Res.

[CR28] Mulyadi CA, Harahap Y, Muliawan HS (2023). Comparison of microsampling and conventional sampling techniques for quantification of warfarin in blood samples: a systematic review. Pharm Sci.

[CR29] Nakayama SMM, Morita A, Ikenaka Y, Mizukawa H, Ishizuka M (2019). A review: poisoning by anticoagulant rodenticides in non-target animals globally. J Vet Med Sci.

[CR30] Nidheesh PV, Gandhimathi R (2012). Trends in electro-Fenton process for water and wastewater treatment: an overview. Desalination.

[CR31] Pignatello JJ, Oliveros E, MacKay A (2006). Advanced oxidation processes for organic contaminant destruction based on the fenton reaction and related chemistry. Crit Rev Environ Sci Technol.

[CR32] Rattner BA, Lazarus RS, Elliott JE, Shore RF, van den Brink N (2014). Adverse outcome pathway and risks of anticoagulant rodenticides to predatory wildlife. Environ Sci Technol.

[CR33] Regnery J, Friesen A, Geduhn A, Göckener B, Kotthoff M, Parrhysius P, Brinke M (2018). Rating the risks of anticoagulant rodenticides in the aquatic environment: a review. Environ Chem Lett.

[CR34] Regnery J, Parrhysius P, Schulz RS, Mohlenkamp C, Buchmeier G, Reifferscheid G, Brinke M (2019). Wastewater-borne exposure of limnic fish to anticoagulant rodenticides. Water Res.

[CR35] Regnery J, Schulz RS, Parrhysius P, Bachtin J, Brinke M, Schafer S, Friesen A (2020). Heavy rainfall provokes anticoagulant rodenticides’ release from baited sewer systems and outdoor surfaces into receiving streams. Sci Total Environ.

[CR36] Rettie AE, Korzekwa KR, Kunze KL, Lawrence RF, Eddy AC, Aoyama T, Trager WF (1992). Hydroxylation of Warfarin by human cDNA-expressed cytochrome P-450: a role for P-4502C9 in the etiology of (S)-warfarin-drug interactions. Chem Res Toxicol.

[CR37] Riha M, Karlickova J, Filipsky T, Jahodar L, Hrdina R, Mladenka P (2014). In vitro copper-chelating properties of flavonoids. Free Radic Biol Med.

[CR38] Rishavy MA, Usubalieva A, Hallgren KW, Berkner KL (2011). Novel insight into the mechanism of the vitamin K oxidoreductase (VKOR): electron relay through Cys43 and Cys51 reduces VKOR to allow vitamin K reduction and facilitation of vitamin K-dependent protein carboxylation. J Biol Chem.

[CR39] Saengtienchai A, Ikenaka Y, Watanabe K, Ishida T, Ishizuka M (2011). Comparative metabolism of warfarin in rats and chickens. Poult Sci.

[CR40] Saito-Shida S, Nemoto S, Matsuda R, Akiyama H (2016). Simultaneous determination of seven anticoagulant rodenticides in agricultural products by gel permeation chromatography and liquid chromatography-tandem mass spectrometry. J Environ Sci Health B.

[CR41] Sato S (2005) Coumarin rodenticides. In Coumarin Rodenticides. In: Drugs and Poisons in Humans (pp. 599–608): Springer Berlin Heidelberg.

[CR42] Sharma A, Ahmad J, Flora SJS (2018). Application of advanced oxidation processes and toxicity assessment of transformation products. Environ Res.

[CR43] Spink DC, Aldous KM, Kaminsky LS (1989). Analysis of oxidative warfarin metabolites by thermospray high-performance liquid chromatography/mass spectrometry. Anal Biochem.

[CR44] Stafford DW (2005). The vitamin K cycle. J Thromb Haemost.

[CR45] Waddell LS, Poppenga RH, Drobatz KJ (2013). Anticoagulant rodenticide screening in dogs: 123 cases (1996–2003). J Am Vet Med Assoc.

[CR46] Wang NN, Zheng T, Zhang GS, Wang P (2016). A review on Fenton-like processes for organic wastewater treatment. J Environ Chem Eng.

[CR47] Wardrop D, Keeling D (2008). The story of the discovery of heparin and warfarin. Br J Haematol.

[CR48] Watanabe KP, Kawata M, Ikenaka Y, Nakayama SM, Ishii C, Darwish WS, Ishizuka M (2015). Cytochrome P450-mediated warfarin metabolic ability is not a critical determinant of warfarin sensitivity in avian species: in vitro assays in several birds and in vivo assays in chicken. Environ Toxicol Chem.

[CR49] Zhang MH, Dong H, Zhao L, Wang DX, Meng D (2019). A review on Fenton process for organic wastewater treatment based on optimization perspective. Sci Total Environ.

