# Supplementary material for: Developing a GC-EI-MS/MS method for quantifying warfarin and five hydroxylated metabolites generated by the Fenton reaction
Source: Environ Sci Pollut Res Int. 2024 Feb 8;31(11):16986–94. doi: 10.1007/s11356-024-32133-3 (PMC10894117; doi:10.1007/s11356-024-32133-3)
Supplement: Supplementary file 1 — Supplementary file1 (PDF 1.62 MB) [file 11356_2024_32133_MOESM1_ESM.pdf]

Supporting Information

Developing a GC-EI-MS/MS Method for Quantifying Warfarin and Five Hydroxylated Metabolites Generated by the Fenton Reaction.

Wipert Jannik von Törne[1,2], Urszula-Anna Klyk-Seitz[1], Christian Piechotta[1,2]

[1] Bundesanstalt für Materialforschung und –prüfung (BAM), Richard-Willstätter-Straße 11, 12489 Berlin, Germany.
[2] Technischen Universität Berlin, Straße des 17. Juni 135, 10623 Berlin, Germany

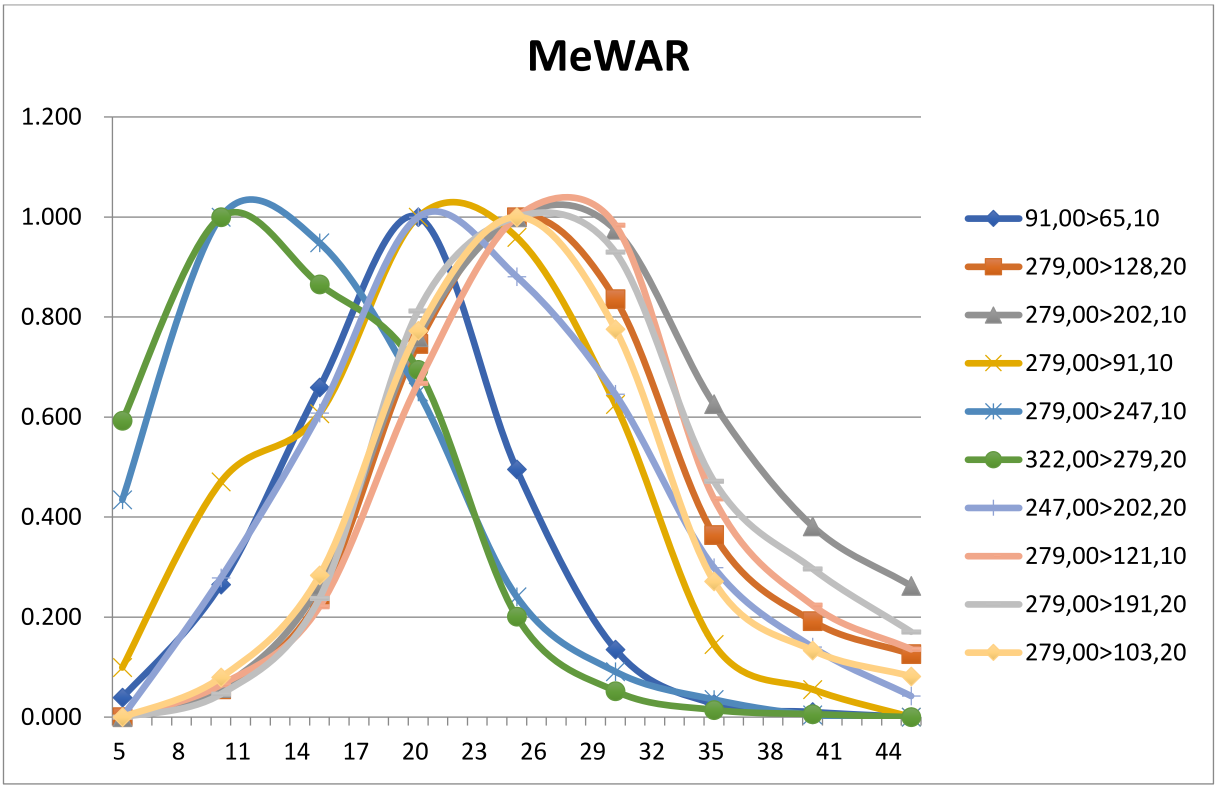

Figure 1: The diagram illustrates the optimization process of transitions and collision energies (CE) of MeWAR.

Table 1: The table highlights the most intense transitions and corresponding collision energies (CE) of the selected product ions of MeWAR.

Product m/z	*91,00>65,10*	*279,00>128,20*	*279,00>202,10*	*279,00>91,10*	*279,00>247,10*	*322,00>279,20*	*247,00>202,20*	*279,00>121,10*	*279,00>191,20*	*279,00>103,20*
Max Int.	122666	109638	74415	70075	59278	57930	54325	49033	29622	26413
CE	20	25	25	20	10	10	20	25	25	25

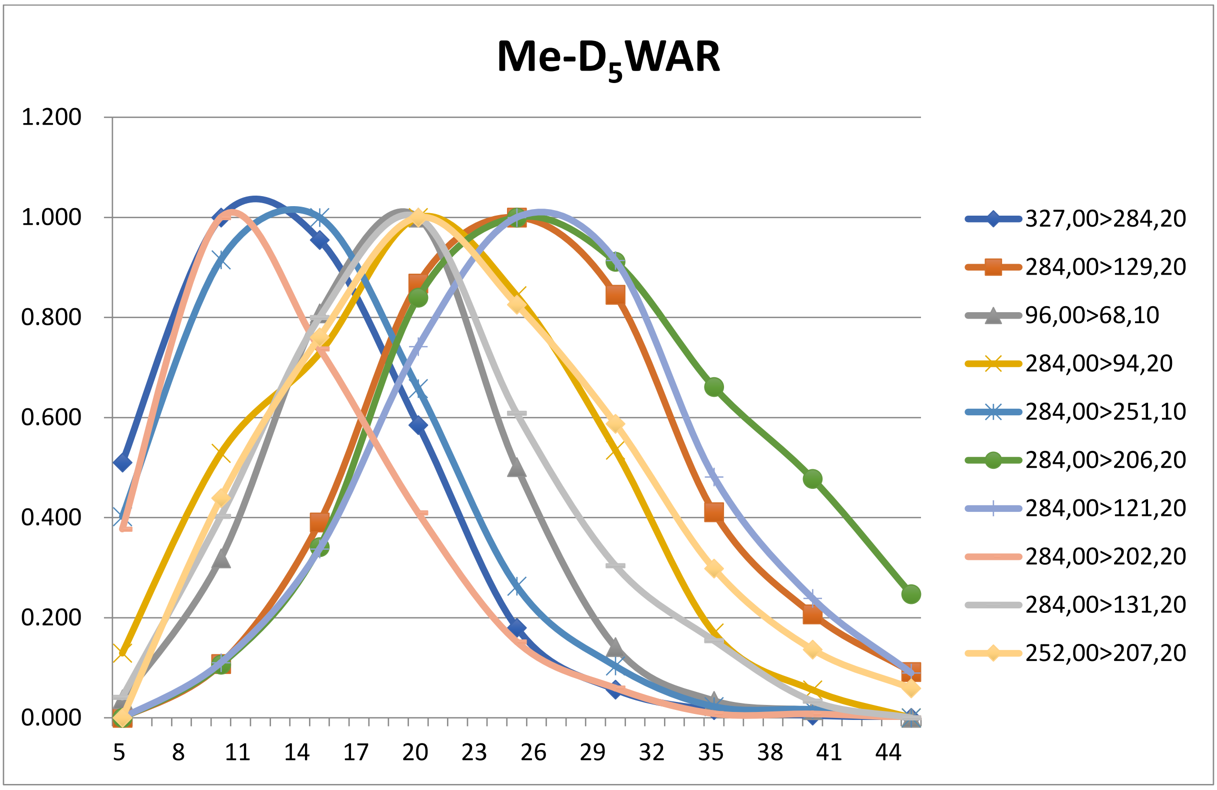

Figure 2: The diagram illustrates the optimization process of transitions and collision energies (CE) of Me-D$_5$WAR.

Table 2: The table highlights the most intense transitions and corresponding collision energies (CE) of the selected product ions of Me-D$_5$WAR.

Product m/z	*327,00>284,20*	*284,00>129,20*	*96,00>68,10*	*284,00>94,20*	*284,00>251,10*	*284,00>206,20*	*284,00>121,20*	*284,00>202,20*	*284,00>131,20*	*252,00>207,20*
**Max Int.**	68807	60464	42341	32411	28396	28098	26677	24323	16188	14699
**CE**	10	25	20	20	15	25	25	10	20	20

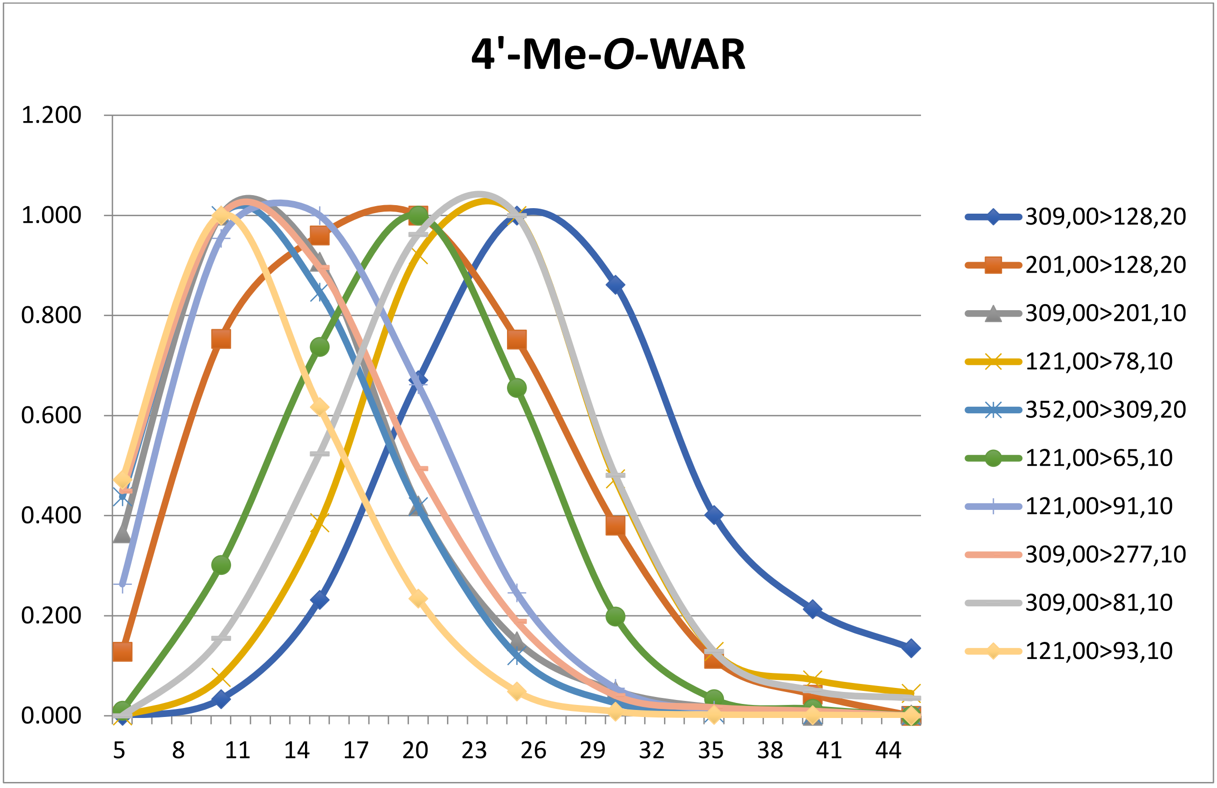

Figure 3: The diagram illustrates the optimization process of transitions and collision energies (CE) of 4'-Me-*O*-WAR.

Table 3: The table highlights the most intense transitions and corresponding collision energies (CE) of the selected product ions of 4'-Me-*O*-WAR.

Product m/z	*309,00>128,20*	*201,00>128,20*	*309,00>201,10*	*121,00>78,10*	*352,00>309,20*	*121,00>65,10*	*121,00>91,10*	*309,00>277,10*	*309,00>81,10*	*121,00>93,10*
Max Int.	123039	102346	98897	92680	59191	58337	47699	36422	32965	31657
CE	25	20	10	25	10	20	15	10	25	10

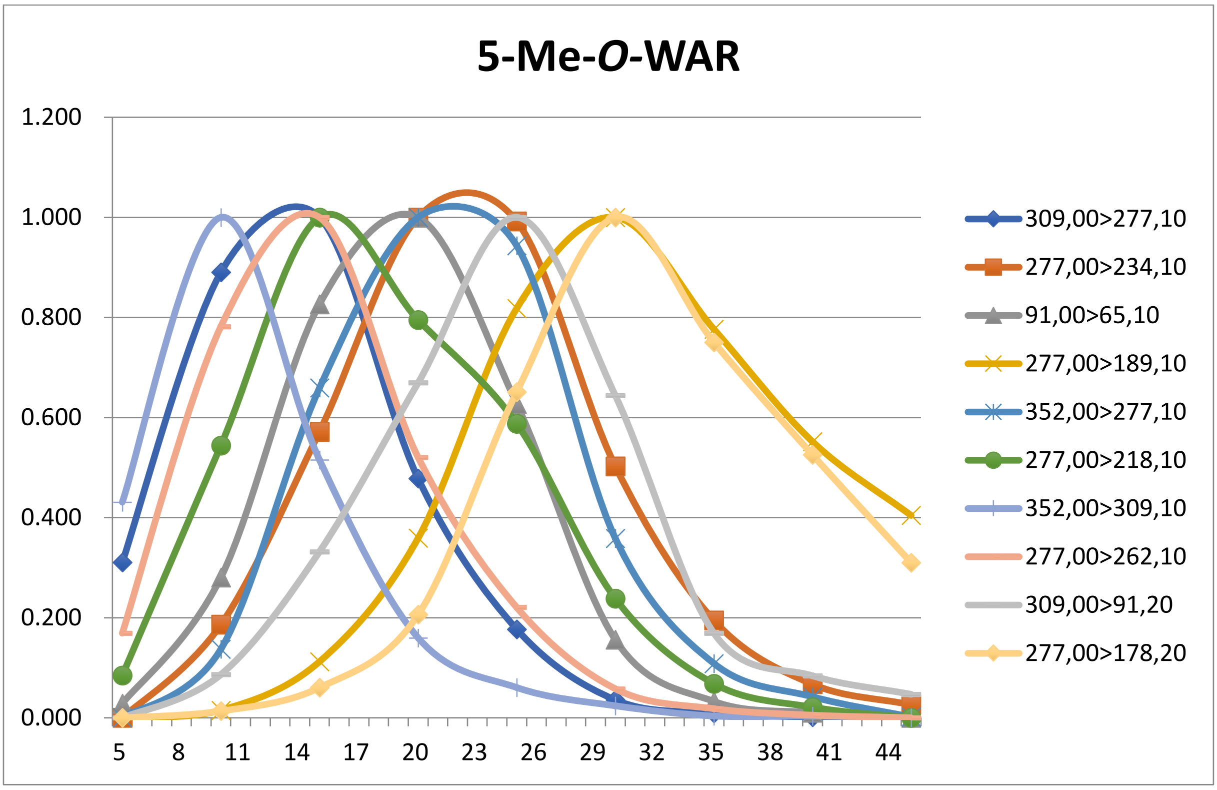

Figure 4: The diagram illustrates the optimization process of transitions and collision energies (CE) of 5-Me-*O*-WAR.

Table 4: The table highlights the most intense transitions and corresponding collision energies (CE) of the selected product ions of 5-Me-*O*-WAR.

Product m/z	*309,00>277,10*	*277,00>234,10*	*91,00>65,10*	*277,00>189,10*	*352,00>277,10*	*277,00>218,10*	*352,00>309,10*	*277,00>262,10*	*309,00>91,20*	*277,00>178,20*
Max Int.	88895	61869	57401	38010	32181	31163	28992	27184	23632	21034
CE	15	20	20	30	20	15	10	15	25	30

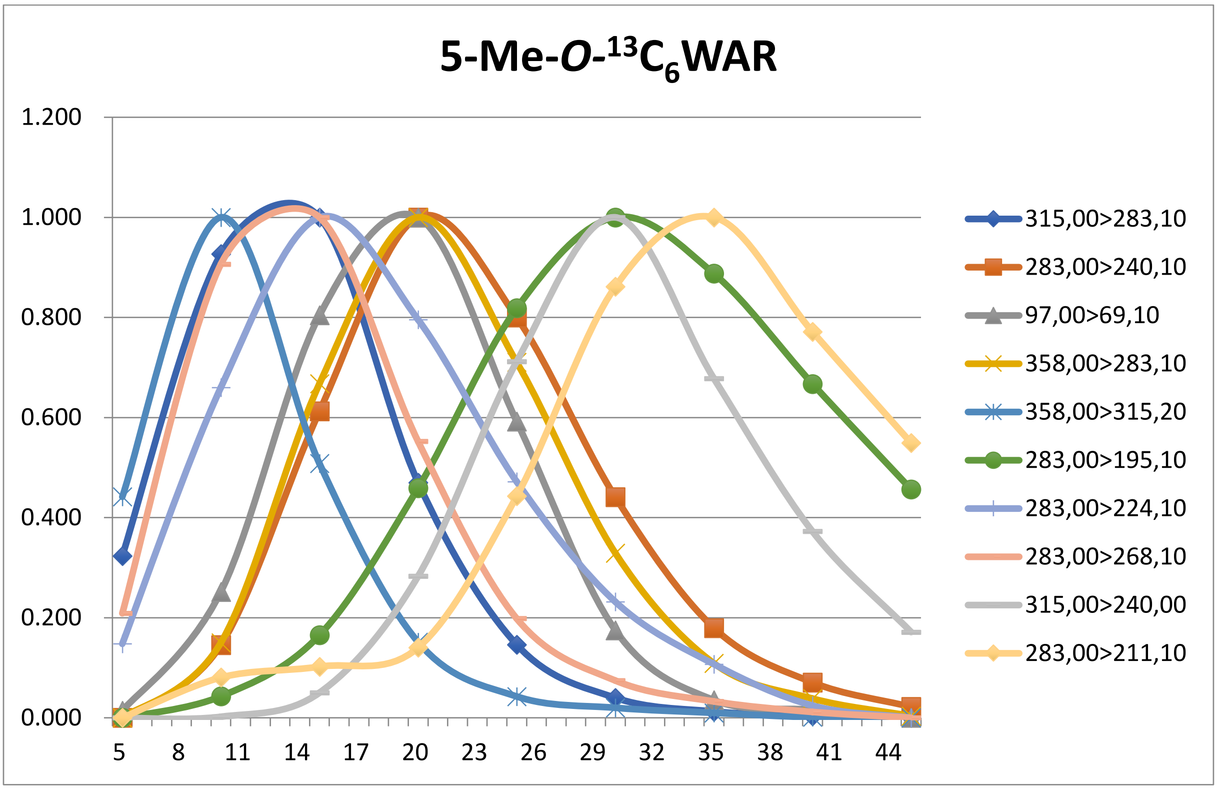

Figure 5: The diagram illustrates the optimization process of transitions and collision energies (CE) of 5-Me-*O*-$^{13}C_6$WAR.

Table 5: The table highlights the most intense transitions and corresponding collision energies (CE) of the selected product ions of 5-Me-*O*-$^{13}C_6$WAR.

Product m/z	*315,00>283,10*	*283,00>240,10*	*97,00>69,10*	*358,00>283,10*	*358,00>315,20*	*283,00>195,10*	*283,00>224,10*	*283,00>268,10*	*315,00>240,00*	*283,00>211,10*
Max Int.	124908	89129	52040	49395	44096	42889	40949	36264	27755	25787
CE	15	20	20	20	10	30	15	15	30	35

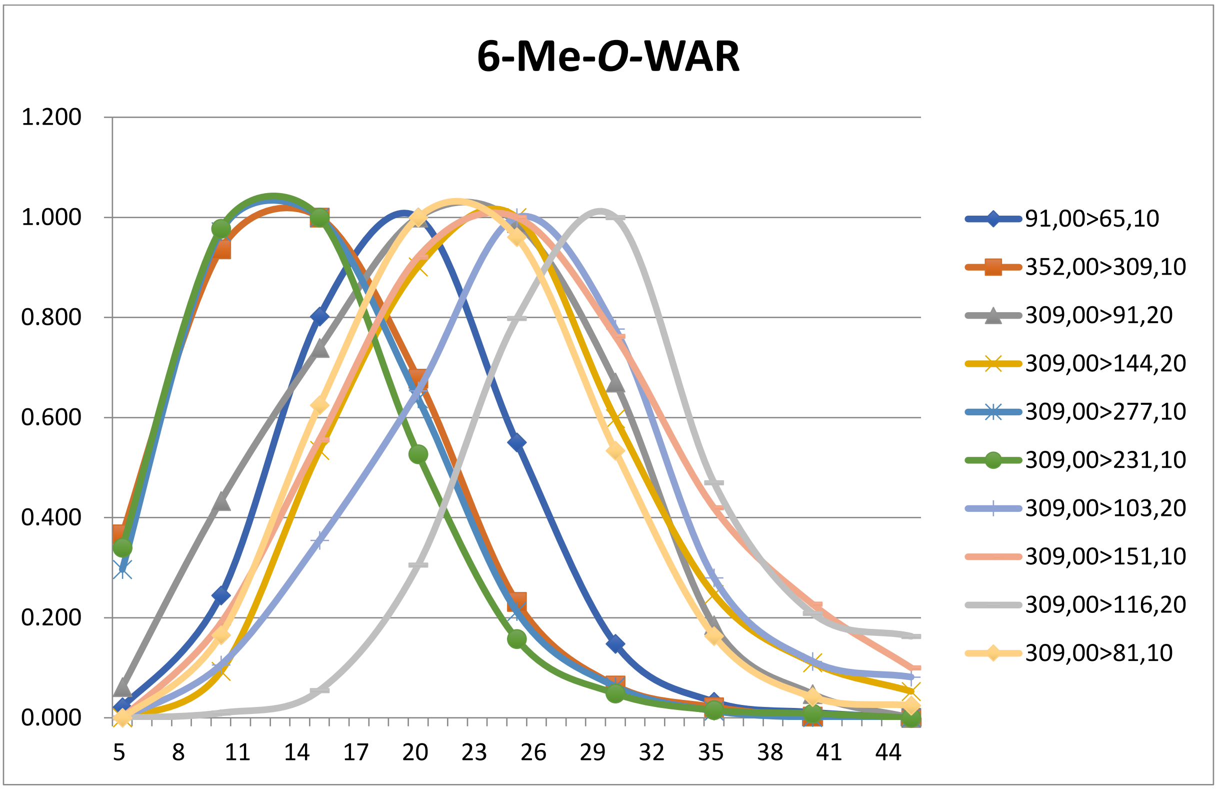

Figure 6: The diagram illustrates the optimization process of transitions and collision energies (CE) of 6-Me-*O*-WAR.

Table 6: The table highlights the most intense transitions and corresponding collision energies (CE) of the selected product ions of 6-Me-*O*-WAR.

Product m/z	*91,00>65,10*	*352,00>309,10*	*309,00>91,20*	*309,00>144,20*	*309,00>277,10*	*309,00>231,10*	*309,00>103,20*	*309,00>151,10*	*309,00>116,20*	*309,00>81,10*
Max Int.	65602	56684	47573	43408	40859	25407	19123	19102	17949	15305
CE	20	15	20	25	15	15	25	25	30	20

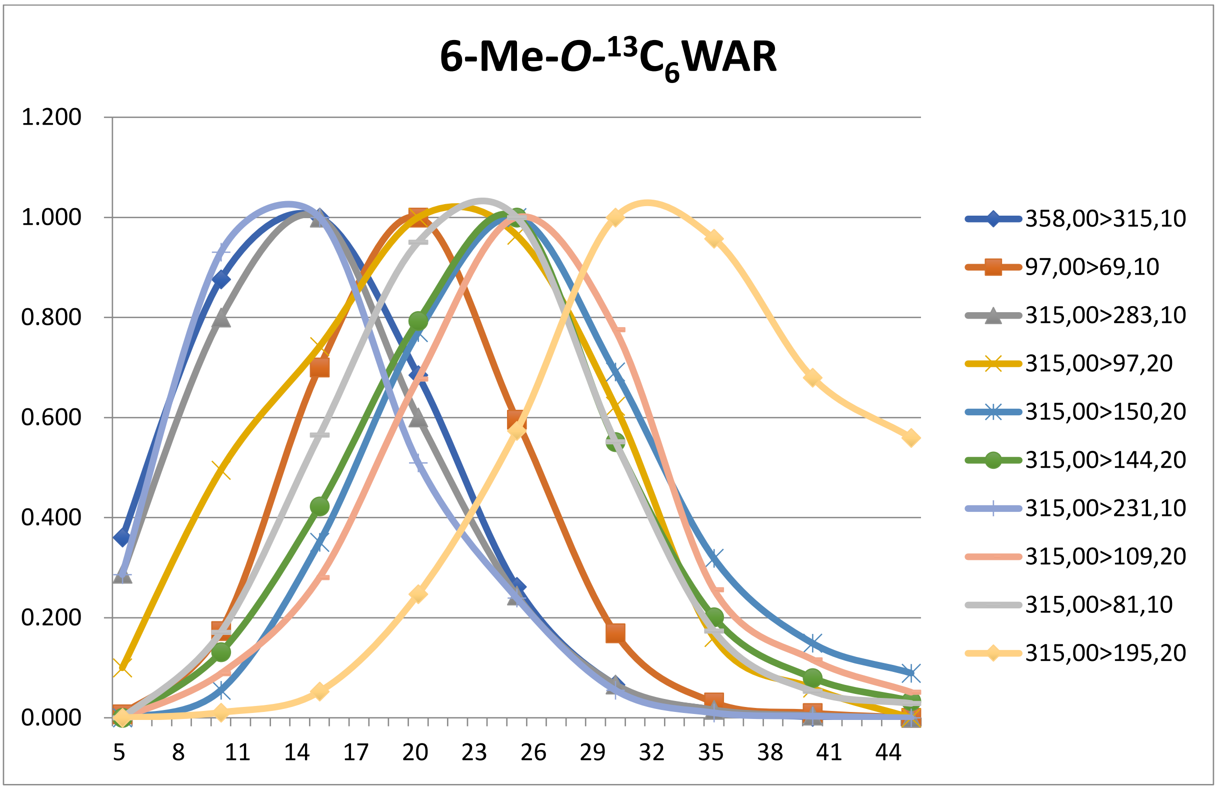

Figure 7: The diagram illustrates the optimization process of transitions and collision energies (CE) of 6-Me-*O*-$^{13}C_6$WAR.

Table 7: The table highlights the most intense transitions and corresponding collision energies (CE) of the selected product ions of 6-Me-*O*-$^{13}C_6$WAR.

Product m/z	*358,00>315,10*	*97,00>69,10*	*315,00>283,10*	*315,00>97,20*	*315,00>150,20*	*315,00>144,20*	*315,00>231,10*	*315,00>109,20*	*315,00>81,10*	*315,00>195,20*
Max Int.	53608	41283	37556	37321	25383	23849	22873	21890	14682	12808
CE	15	20	15	20	25	25	15	25	25	30

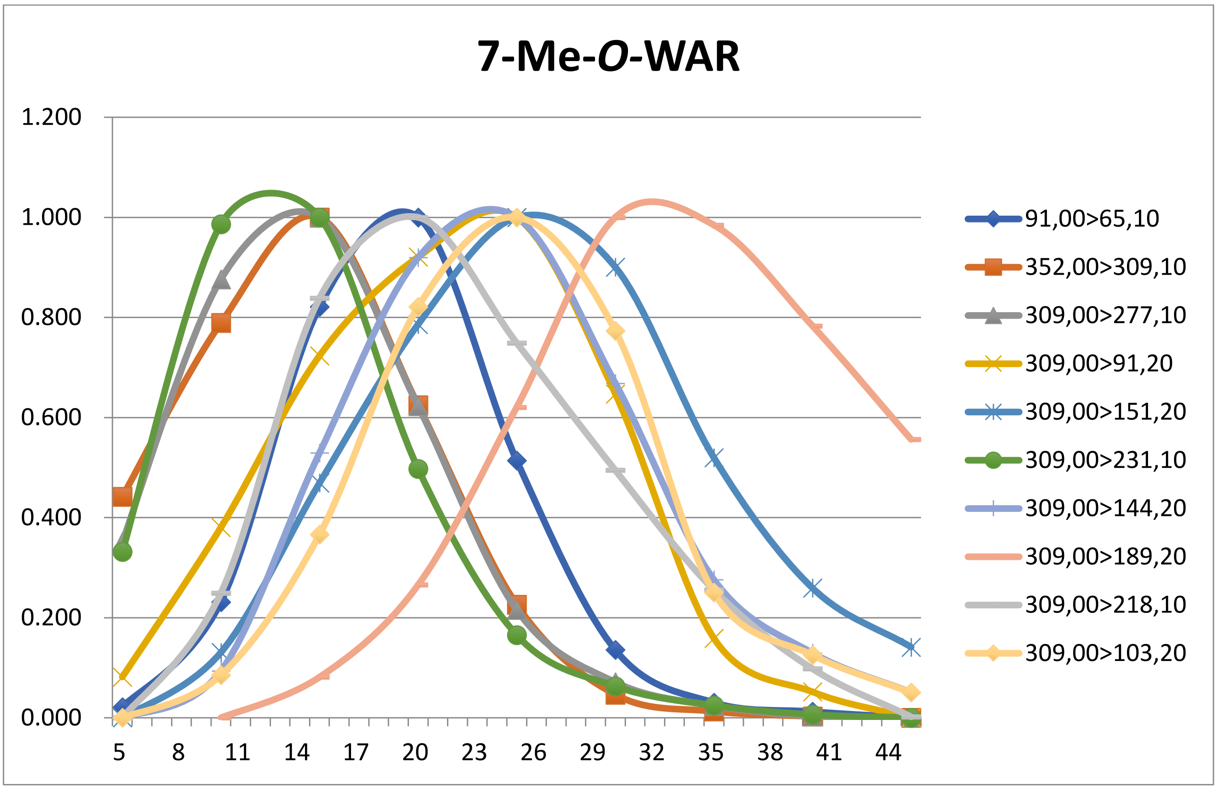

Figure 8: The diagram illustrates the optimization process of transitions and collision energies (CE) of 7-Me-*O*-WAR.

Table 8: The table highlights the most intense transitions and corresponding collision energies (CE) of the selected product ions of 7-Me-*O*-WAR.

Product m/z	*91,00>65,10*	*352,00>309,10*	*309,00>277,10*	*309,00>91,20*	*309,00>151,20*	*309,00>231,10*	*309,00>144,20*	*309,00>189,20*	*309,00>218,10*	*309,00>103,20*
Max Int.	104505	61602	48865	42728	38084	36073	35661	27305	23175	17802
CE	20	15	15	25	25	15	25	30	20	25

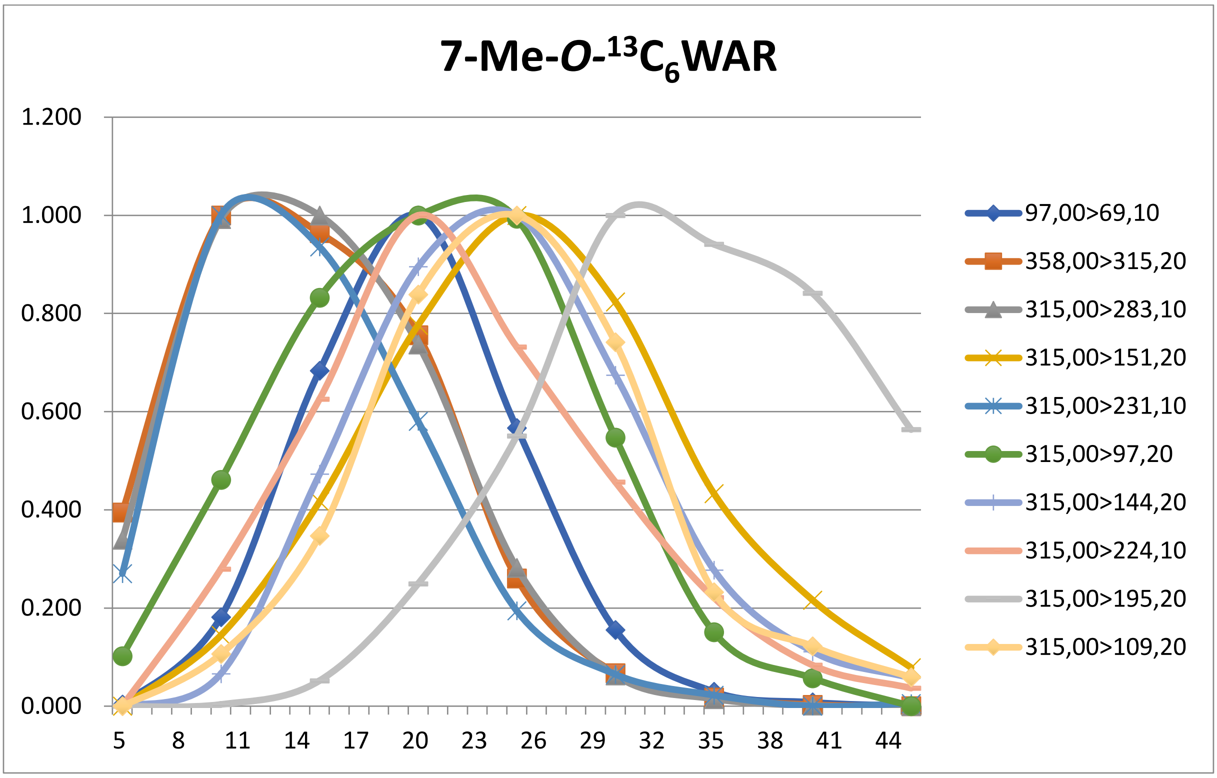

Figure 9: The diagram illustrates the optimization process of transitions and collision energies (CE) of 7-Me-*O*-$^{13}C_6$WAR.

Table 9: The table highlights the most intense transitions and corresponding collision energies (CE) of the selected product ions of 7-Me-*O*-$^{13}C_6$WAR.

Product m/z	*97,00>69,10*	*358,00>315,20*	*315,00>283,10*	*315,00>151,20*	*315,00>231,10*	*315,00>97,20*	*315,00>144,20*	*315,00>224,10*	*315,00>195,20*	*315,00>109,20*
Max Int.	53489	42233	32544	28731	26868	24846	20346	18131	17163	12597
CE	20	10	15	25	10	20	25	20	30	25

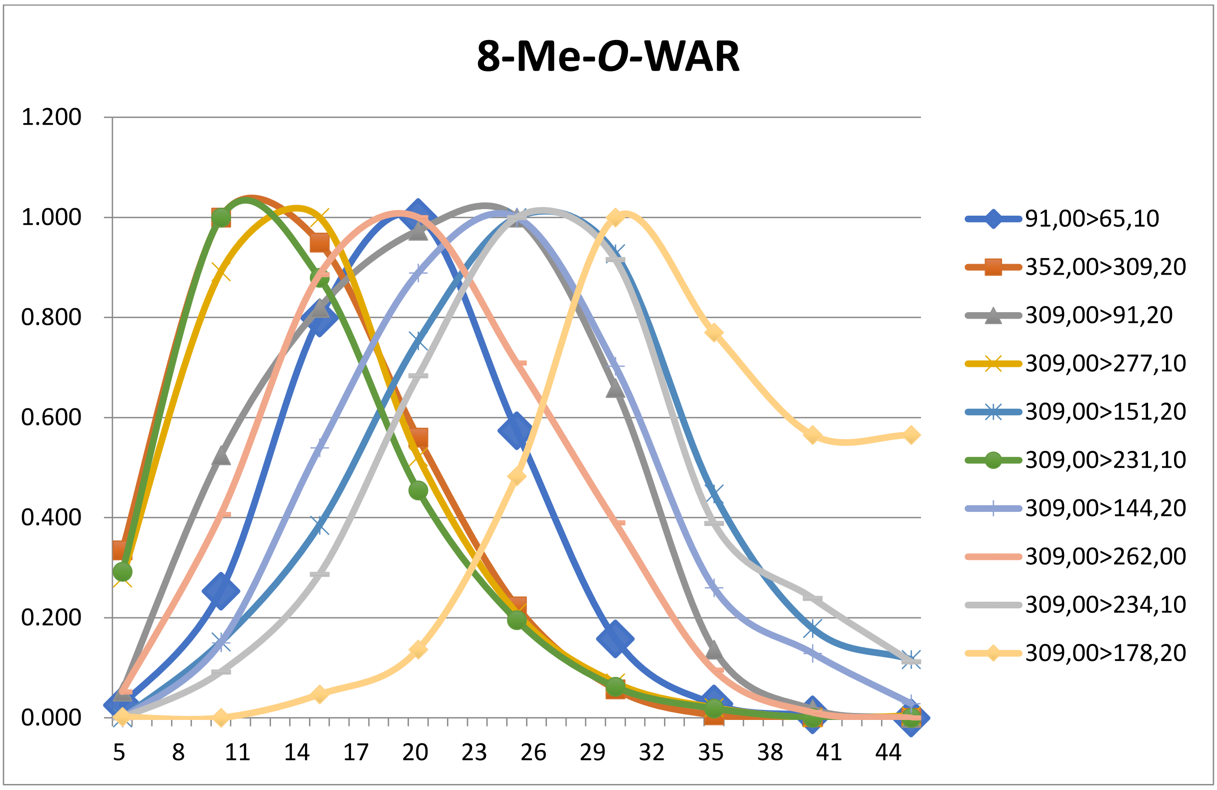

Figure 10: The diagram illustrates the optimization process of transitions and collision energies (CE) of 8-Me-*O*-WAR.

Table 10: The table highlights the most intense transitions and corresponding collision energies (CE) of the selected product ions of 8-Me-*O*-WAR.

Product m/z	*91,00>65,10*	*352,00>309,20*	*309,00>91,20*	*309,00>277,10*	*309,00>151,20*	*309,00>231,10*	*309,00>144,20*	*309,00>262,00*	*309,00>234,10*	*309,00>178,20*
Max Int.	46814	34938	31225	28350	20320	16053	11732	10665	10225	9404
CE	20	10	25	15	25	10	25	20	25	30
